# An *in vitro* experimental investigation of oscillatory flow in the cerebral aqueduct

**DOI:** 10.1016/j.euromechflu.2024.01.010

**Published:** 2024-01-23

**Authors:** S. Sincomb, F. Moral-Pulido, O. Campos, C. Martínez-Bazán, V. Haughton, A.L. Sánchez

**Affiliations:** a Department of Aerospace and Mechanical Engineering, University of California, San Diego, La Jolla, 92093-0411, CA, USA; b Department of Mechanical and Mining Engineering, University of Jaen, Jaen, 23071, Spain; c Andalusian Institute for Earth System Research, University of Jaen, Jaen, 23071, Spain; d Department of Mechanics of Structures and Hydraulic Engineering, University of Granada, Granada, 18001, Spain; e Andalusian Institute for Earth System Research, University of Granada, Granada, 18006, Spain; f School of Medicine and Public Health, University of Wisconsin-Madison, Madison, 53706, WI, USA

**Keywords:** Cerebrospinal fluid, Cerebral aqueduct, Interventricular pressure

## Abstract

This *in vitro* study aims at clarifying the relation between the oscillatory flow of cerebrospinal fluid (CSF) in the cerebral aqueduct, a narrow conduit connecting the third and fourth ventricles, and the corresponding interventricular pressure difference. Dimensional analysis is used in designing an anatomically correct scaled model of the aqueduct flow, with physical similarity maintained by adjusting the flow frequency and the properties of the working fluid. The time-varying pressure difference across the aqueduct corresponding to a given oscillatory flow rate is measured in parametric ranges covering the range of flow conditions commonly encountered in healthy subjects. Parametric dependences are delineated for the time-averaged pressure fluctuations and for the phase lag between the transaqueductal pressure difference and the flow rate, both having clinical relevance. The results are validated through comparisons with predictions obtained with a previously derived computational model. The parametric quantification in this study enables the derivation of a simple formula for the relation between the transaqueductal pressure and the stroke volume. This relationship can be useful in the quantification of transmantle pressure differences based on non-invasive magnetic-resonance-velocimetry measurements of aqueduct flow for investigation of CSF-related disorders.

## 1. Introduction

The cerebrospinal fluid (CSF) fills the cerebral ventricles and the subarachnoid space (SAS) surrounding the brain and the spinal cord. Its motion has been studied and modeled extensively [1,2], partly because of its role in key physiological functions associated with the transport of hormones, nutrients, and neuroendocrine substances [3–5] and partly because of its involvement in the development of different pathological conditions related to abnormal flow behavior, such as normal pressure hydrocephalus (NPH) [6,7].

Slow net flow of CSF occurs between the ventricles, where it is primarily produced in the choroid plexus, and the SAS, from which it exists via multiple pathways [8,9]. Besides this slow motion, there exists a much faster oscillatory motion driven by the spatial pressure differences induced by the cardiac and respiratory cycles [1,2,10–12]. In the cranial cavity, the largest velocities are found in the cerebral aqueduct, which is a narrow passage of length L~10-15mm and slowly varying cross-sectional area $A\sim 3-7{\;mm}^{2}$ connecting the third and fourth ventricles [1,13]. Its nearly cylindrical shape [14] can be described by assuming a circular section with radius $a=\sqrt{A/\pi }\sim 1-1.5\;mm$. CSF velocities in the aqueduct have clinical potential value in investigating the development of NPH [6,15] and understanding the effects of different treatments like shunting [16] or lumbar puncture [17].

A closely related parameter of clinical interest is the transmantle pressure, or instantaneous pressure difference between the lateral ventricles and the cerebral SAS. It is known that the largest pressure drop occurs across the aqueduct [18,19] and therefore, to a good approximation, one can use the transaqueductal pressure difference, i.e. the pressure difference between the third and fourth ventricles $\Delta p(t)=p_{3}-p_{4}$, to characterize the transmantle pressure difference. The value of Δp(t) fluctuates from positive to negative about a negligibly small time-averaged value [20]. The pressure fluctuations, on the order of a few pascals, drive the oscillatory flow across the aqueduct, with associated volumetric rates Q(t) having peak values on the order of 0.1 mL/s [21–23].

The transmantle pressure has been argued to play a role in the ventricle enlargement characterizing NPH patients [24]. Although the mean transmantle force exerted on the brain is very small [20], it has been hypothesized that the cyclic strain field acting on the brain parenchyma over a long time can explain observed changes in brain viscoelastic properties in NPH patients with altered CSF flow dynamics [25]. The phase lag between Δp(t) and the flow rate Q(t) has also been investigated because of its potential clinical relevance in NPH patients [26], thereby underscoring the implications of the wave forms involved in aqueduct flow.

Direct measurements of the transmantle pressure require accurate simultaneous readings from two separate high-resolution pressure sensors [27], an invasive procedure with considerable risk factors [28]. Since the interventricular pressure difference Δp(t) drives the flow in the aqueduct, its value can be inferred from noninvasive magnetic resonance imaging (MRI) measurements of the oscillatory aqueduct flow rate Q(t) [19]. Pressure differences associated with the cardiac-driven flow are about four times larger than those associated with respiration [27]. Different models have been developed for the relation between Δp(t) and Q(t) [19,29,30], needed to enable estimates of the former from measurements of the latter [31]. As shown previously [30], accurate predictions require consideration of effects of flow acceleration as well as pressure losses at the nearly inviscid entrance regions connecting the aqueduct with the ventricles.

*In vivo* investigations of aqueduct flow using MRI techniques have been instrumental in quantifying associated flow rates and stroke volumes [see, e.g. 10,12, and references therein]. More detailed quantitative information has been acquired in different computational investigations [14,32–34] and also in *in vitro* experimental studies of aqueduct flow [35–38]. These *in vitro* investigations have examined, in particular, the flow in the third ventricle of anatomically correct models, using either particle tracking velocimetry (PTV) combined with phase contrast (PC) MRI [35] or particle image velocimetry (PIV) [36], the latter study pertaining to steady flow, which is not representative of the markedly unsteady conditions found in the human aqueduct, for which resulting Strouhal numbers are of order unity [30]. As in the present investigation, the phantom study of Schibli et al. [35] uses a scaled-up model, with physical similarity accounted for by modifying the properties of the working fluid. Their analysis, imposing a pressure difference varying sinusoidally in time, identified many interesting features pertaining to the unsteady flow in the third ventricle. These early studies, specifically targeting the ventricular flow structure, were followed by the *in vitro* study of Bottan et al. [37], who, by using a life-size model of the entire cranial domain, were able to provide simultaneous measurements of the intracranial pressure fluctuation, aqueduct pressure drop, and aqueduct flow rate driven by the cardiac cycle. Their phantom model, with clear potential for extensive measurements of physiologically relevant quantities, was used to generate a single set of representative waveforms, yielding shapes that did not fully match those obtained on healthy subjects [37], departures being attributed to limitations in the bandwidth of the actuation system. In a more recent study, Holmlund et al. [38] performed benchtop aqueduct experiments of unsteady flow in a simplified aqueduct model with a realistic flow rate driven by a syringe pump. Corresponding interventricular pressure differences were measured and associated time-averaged values of about 0.5 Pa were computed, but corresponding pressure fluctuations and relative flow-rate/pressure-gradient phase lags, both of clinical interest, were not reported.

The purpose of the present *in vitro* experiments is that of complementing these previous studies by providing a more complete parametric description of the interventricular pressure difference for a range of flow conditions found in healthy subjects. Experiments were conducted with an anatomically correct model, conveniently scaled up by a factor of 10, as needed to enable pressure measurements to be made, with care taken to maintain physical similarity. While most experiments considered a realistic time-dependent flow rate Q(t), to investigate influences of canal shape and flow-rate waveform an initial set of measurements used instead a sinusoidal flow rate in an aqueduct model of constant circular section. The experimental results were validated through comparisons with predictions obtained using the previously published theoretical model [30]. The experimental measurements, presented in dimensionless form, provide a thorough description of the relation between the flow rate and the interventricular pressure difference. The new information can be useful in designing future protocols for quantification of transmantle pressure differences based on non-invasive phase contrast (PC) MRI measurements.

The rest of the paper is organized as follows. The dimensional arguments employed in scaling the experiment are introduced in Section 2. The description of the facility is given in Section 3, followed in Section 4 by a detail account of the data acquisition procedures. Experimental results are presented in Section 5. Finally, conclusions and future extensions of the work are discussed in Section 6.

## 2. Geometrical and physical similarity

One could envision an experiment mimicking exactly the cerebral aqueduct conditions. Water would be used as working fluid, since its density $\rho =1000\;kg/m^{3}$ and kinematic viscosity $v=0.71\times 10^{-6}m^{2}/s$ differ from those of CSF by a negligibly small amount. The setup would involve a tiny canal whose shape and size would have to be identical to those of the human aqueduct (i.e. L≃10-15mm and $a=\sqrt{A/\pi }≃1-1.5\;mm$), a micro-pump able to deliver oscillating flow rates with peak values on the order of 0.1 mL/s, and a sensor with the capability of measuring pressure differences of a few pascals with sufficient accuracy. Of course, such an experiment would be difficult due to microfabrication challenges and also because the devices that are required to deliver/measure accurately such small flow rates/pressure differences are uneconomical. The alternative procedure followed here involves scaling up the aqueduct model as well as the resulting flow rate and pressure difference, in such a way that the experiment can be performed in the lab using the available resources.

A phantom model of the aqueduct with a size scaling factor χ=10 was built to investigate experimentally the relation between the aqueduct flow rate and its associated interventricular pressure drop. In scaling the model, account was taken of the fact that, to guarantee that the results of the experiment represent the flow in the human aqueduct, the experimental conditions must be physically similar to those found in the human aqueduct, in that all dimensionless governing parameters must take the same values in the experiment and the human brain. In reducing the parametric dependence of the interventricular pressure Δp(t) we began by noting that the oscillatory flow depends on the canal shape, including its length L and mean radius

(1) $$\overset{ˆ}{a}=\frac{1}{L}\int_{0}^{L}\;\sqrt{A(x)/\pi }dx,$$

where x represents the longitudinal distance measured from the fourth ventricle, on the density ρ and kinematic viscosity v of the fluid, on the frequency of the oscillatory motion (given, for example, by the angular frequency ω, related to the period T according to ω=2π/T), and on the stroke volume $V_{s}=\frac{1}{2}\int_{t}^{t+2\pi /\omega }\;|Q|dt$ being displaced back and forth along the canal, which can be alternatively represented by the stroke length $L_{s}=V_{s}/\left(\pi {\overset{ˆ}{a}}^{2}\right)$.

Following our previous analysis [30], we choose to characterize the pressure difference Δp(t) by the mean value of its magnitude

(2) $$\langle \left|\Delta p\right|\rangle =\frac{\omega }{2\pi }\int_{t}^{t+2\pi /\omega }\left|\Delta p\right|\text{d}t,$$

so that we can write

(3) $$\langle \left|\Delta p\right|\rangle =\langle \left|\Delta p\right|\rangle \left(L,\hat{a},\rho ,v,L_{s},\omega \right).$$

Since the functional dependence features six parameters, three of which have independent dimensions, straightforward application of the Buckingham Π theorem affords reduction from six dimensional parameters to three dimensionless parameters, leading to

(4) $$\langle \left|\Pi \right|\rangle =\frac{\langle \left|\Delta p\right|\rangle }{\rho \omega^{2}LL_{s}}=f\left(\frac{\hat{a}}{L},\frac{L_{s}}{L},\alpha \right),$$

where

(5) $$\alpha ={\left(\frac{\omega {\overset{ˆ}{a}}^{2}}{v}\right)}^{1/2}$$

is the Womersley number of the flow. The characteristic value used to scale Δp follows from assuming that the local acceleration $\partial u/\partial t\sim L_{s}\omega^{2}$ is comparable to the pressure force per unit mass $\rho^{-1}\partial p/\partial x$, that being always the case for the flow conditions found in the cerebral aqueduct. While the aqueduct aspect ratio is very large, giving values of $\overset{ˆ}{a}/L$ in the range $1/20≲\overset{ˆ}{a}/L≲1/10$, the dimensionless stroke length $L_{s}/L$ and the Womersley number α are of order unity, with typical values lying in the ranges

(6) $$0.5≲L_{s}/L≲1.5\;\text{and}\;2≲\alpha ≲4.$$

**Fig. 1.**
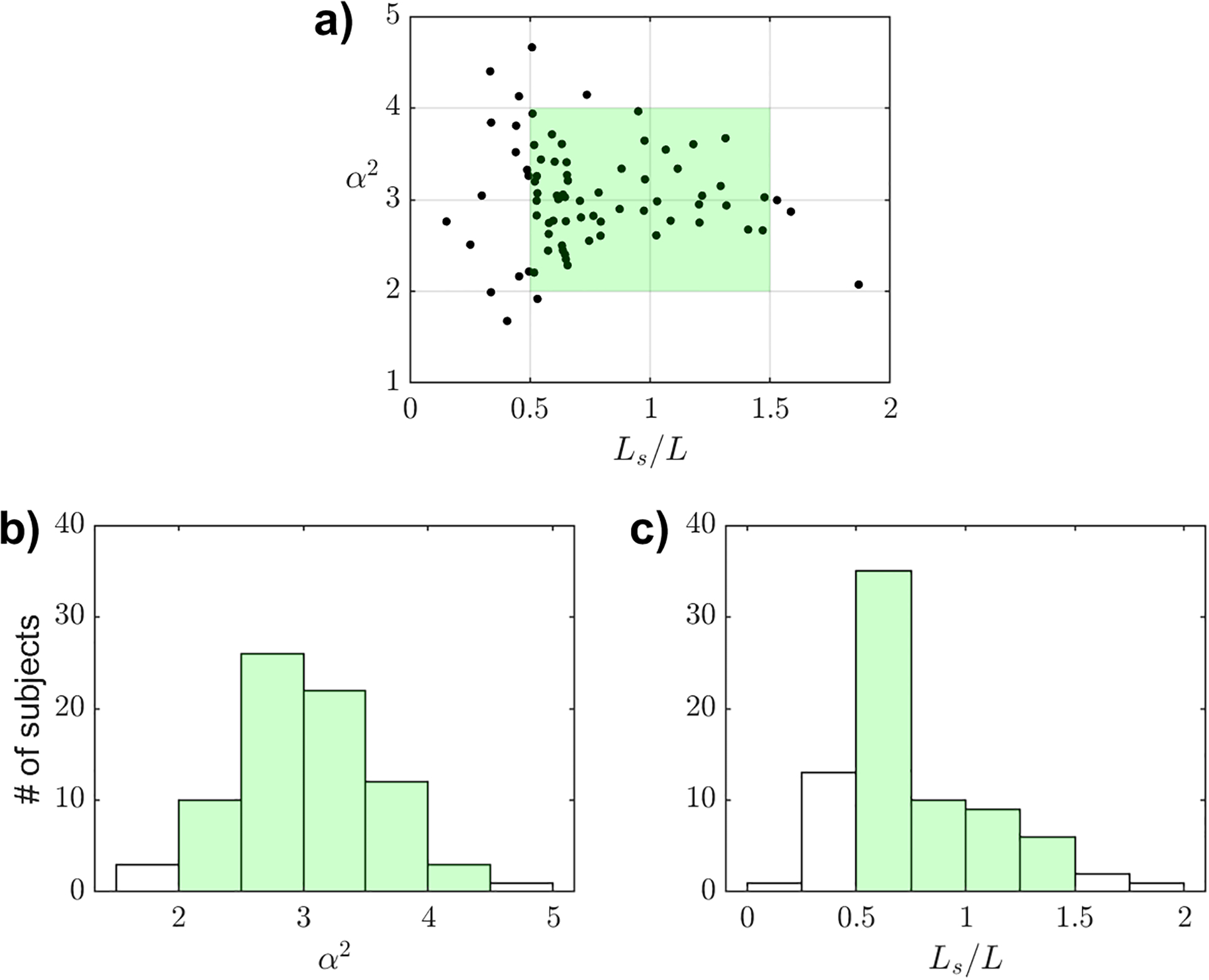
(a) Scatter plot of values of the Womersley number (α) and stroke length-to-aqueduct length ratio $\left(L_{s}/L\right)$ for 77 healthy volunteers participating in an IRB-approved study at HMRI, as reported elsewhere [31]. Distribution of these values for (b) α and (c) $L_{s}/L$.

For illustrative purposes, the specific values of $L_{s}/L$ and α corresponding to the 77 subjects considered in our previous study [31] are shown in Fig. 1. The experiments reported below correspond to α=(2,3,4) with $0.5≲L_{s}/L≲1.5$, thereby covering the conditions most commonly found in healthy human subjects. Regarding the design of the experiment, it is convenient to consider two flow configurations involving canals of different size that are geometrically similar, so that their aspect ratios $\overset{ˆ}{a}/L$ are identical. According to (4), if the values of α and $L_{s}/L$ are equal in both configurations, then the resulting values of the dimensionless pressure-fluctuation amplitude $\langle \left|\Pi \right|\rangle \langle =\left|\Delta p\right|\rangle /\left(\rho \omega^{2}LL_{s}\right)$ would also be equal. Therefore, to guarantee physical similarity, the dimensions, frequency, stroke length, and kinematic viscosity of the experiment (denoted by the subscript E) must be related to those of the human aqueduct (denoted by the subscript H) by

(7) $$\frac{{\overset{ˆ}{a}}_{E}}{L_{E}}=\frac{{\overset{ˆ}{a}}_{H}}{L_{H}},\;\frac{\omega_{E}{\overset{ˆ}{a}}_{E}^{2}}{v_{E}}=\frac{\omega_{H}{\overset{ˆ}{a}}_{H}^{2}}{v_{H}}\;\text{and}\;\frac{L_{s,E}}{L_{E}}=\frac{L_{s,H}}{L_{H}}.$$

When the above relations are satisfied, then the interventricular pressure difference ${\langle \left|\Delta p\right|\rangle }_{H}$ can be computed in terms of the pressure difference ${\langle \left|\Delta p\right|\rangle }_{E}$ measured between the containers (representing the third and fourth ventricles) with use made of

(8) $$\frac{{\langle \left|\Delta p\right|\rangle }_{E}}{\rho_{E}\omega_{E}^{2}L_{E}L_{s,E}}=\frac{{\langle \left|\Delta p\right|\rangle }_{H}}{\rho_{H}\omega_{H}^{2}L_{H}L_{s,H}}.$$

The above expressions were used in scaling up the experiment by a factor $\chi =L_{E}/L_{H}={\overset{ˆ}{a}}_{E}/{\overset{ˆ}{a}}_{H}=10$. In particular, the working fluid was selected to give experimental pressure differences 100Pa≲Δp≲1500Pa well within the operating range ±10 in H20 (±2490 Pa) of the available pressure sensor. The need for a working fluid different from water is apparent when using (7) to write (8) in the form

(9) $$\frac{{\langle \left|\Delta p\right|\rangle }_{E}}{{\langle \left|\Delta p\right|\rangle }_{H}}=\frac{\rho_{E}}{\rho_{H}}{\left(\frac{\omega_{E}}{\omega_{H}}\right)}^{2}\frac{L_{E}}{L_{H}}\frac{L_{s,E}}{L_{s,H}}=\frac{\rho_{E}}{\rho_{H}}{\left(\frac{v_{E}}{v_{H}}\right)}^{2}\chi^{-2}.$$

Clearly, in order for the pressure differential of the experiment with χ=10 to be larger than that of the cerebral aqueduct (i.e. ${\langle \left|\Delta p\right|\rangle }_{H}\;\sim 5\text{Pa}$), the working fluid in the experiment must be significantly more viscous than CSF, thereby motivating the use of mixtures of glycerol and water. Three different glycerol-water mixtures with relative volume contents (84/16, 80/20, 74/26) were employed in the experiments reported below. The mathematical basis for the selection of the mixtures is presented in Appendix. The experiments were conducted in a temperature-controlled room at 21.5 °C, a value that can be used to evaluate the mixture properties, yielding $v_{E}=(10.5,6.82,3.83)\times 10^{-5}m^{2}/s$ and $\rho_{E}=(1225.6,1216.4,1202.2)kg/m^{3}$. To quantify experimental variability stemming from variations of fluid properties, the temperature of the working fluid was monitored during selected experiments. In all instances, the value remained within the narrow range 21.5 ± 1 °C, with associated deviations of kinematic viscosity on the order of 3% across mixtures and smaller deviations of the density on the order of 1%.

## 3. Experimental facility

**Fig. 2.**
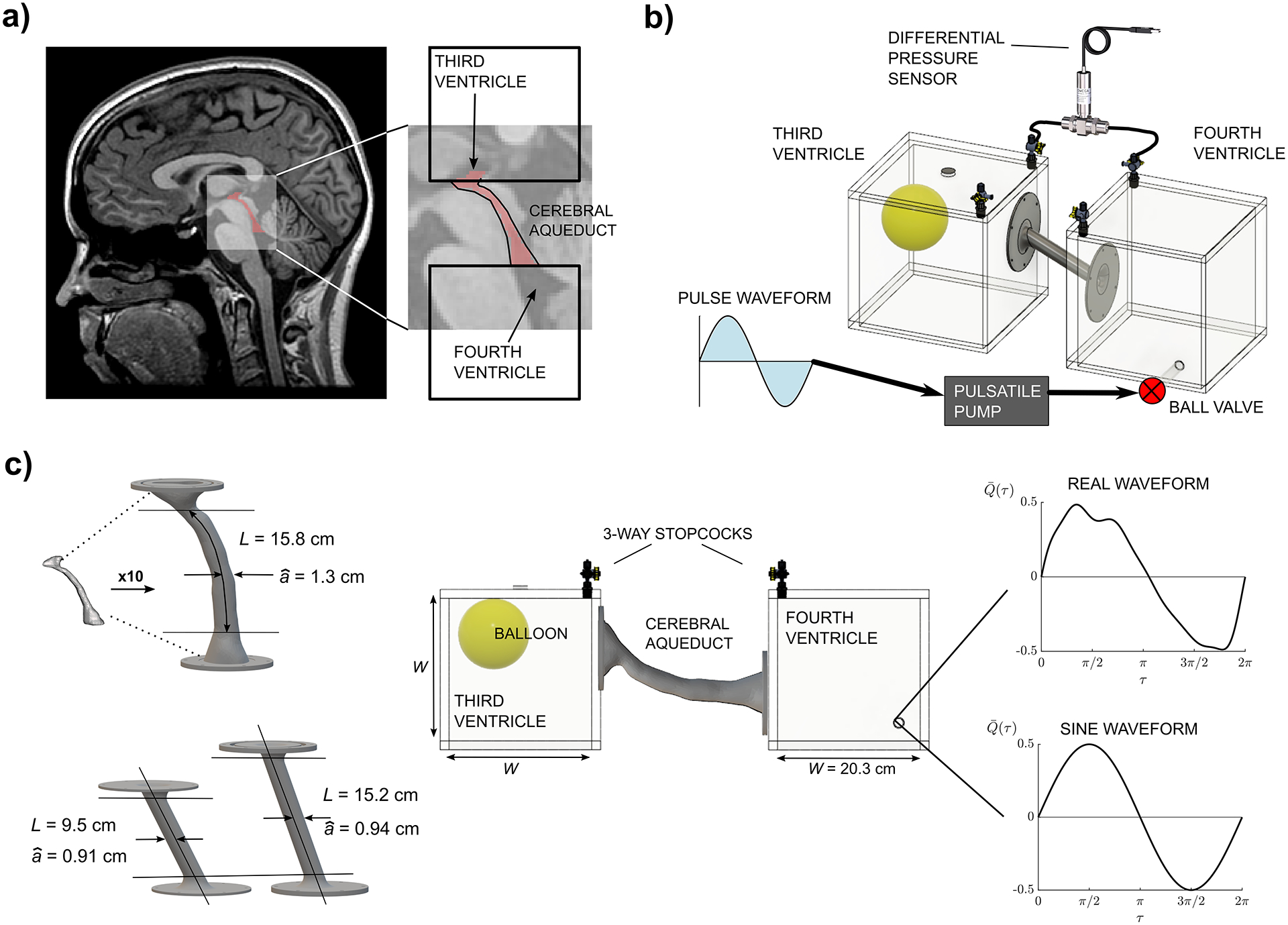
(a) Sagittal MR image of the brain with segmented partial 3rd and 4th ventricles with the aqueduct (red) in the inset. (b) Orthographic view of the experimental setup used to mimic the aqueduct flow. (c) Geometries of the cerebral aqueduct with the corresponding geometrical parameters (real geometry - top left - and canonical geometries - bottom left), side view of the full experimental facility (middle) and experimental flow rate waveforms used as desired output from the pump (sine waveform - bottom right - and MRI waveform - top right).

As shown in the schematics of Fig. 2(b) and 2(c), the *in vitro* model consists of two large reservoirs of cubic shape connected by the cerebral aqueduct model. The reservoirs are constructed using acrylic sheets and assembled with acrylic cement. The periodic flow was generated with a programmable piston pump (SuperPump AR, ViVitro Labs, Victoria, Canada) connected through semi-rigid tubing to a ball valve located near the bottom of one of the reservoirs. The reservoir inner volume of approximately 8 L was sufficiently large to minimize the pressure disturbances introduced by the associated intermittent jet stream. A latex balloon partially filled with air was included in the second reservoir to allow for compliance. It is important to note that, since the flow rate Q(t) is imposed by the peristaltic pump, the specific compliance of the balloon is unimportant for determining experimentally the spatial pressure difference between the two containers, which is only a function of the pulsating motion across the narrow aqueduct. For the same flow rate Q(t), it was verified experimentally that a balloon that is completely filled with air gives pressure fluctuations in both containers, i.e. $p_{3}(t)$ and $p_{4}(t)$, that are much larger than those in an experiment involving a partially filled balloon, but the corresponding instantaneous pressure difference between both containers $\Delta p=p_{3}-p_{4}$ remains unchanged.

A first set of experiments employed the canonical aqueduct model shown in the bottom-left subplot of Fig. 2(c), involving a circular cylinder of uniform radius whose edges were rounded to avoid flow separation during inflow. This simple configuration allowed us to test the dependence of the results on the aspect ratio by using two different tubes with $\overset{ˆ}{a}/L=0.097$ and $\overset{ˆ}{a}/L=0.062$. A second set of experiments employed a realistic model of the cerebral aqueduct, shown in the top-left subplot of Fig. 2(c), corresponding to the subject-specific anatomy used in our previous study [31] (associated IRB approved through Huntington Medical Research Institutes). In characterizing the subject’s anatomy, high-resolution anatomic MR images were used to segment the CSF contained in the aqueduct using ITK-SNAP (Version 3.6.0; www.itksnap.org) [39]; i.e. see Fig. 2(a). The resulting segmentation model was used to determine the variation of the cross-sectional area A(x) with longitudinal distance x, which was then used to calculate the local radius $a(x)=\sqrt{A(x)/\pi }$ of the corresponding circular section and its associated mean value $\overset{ˆ}{a}$, computed according to (1). The result was then scaled by a factor of 10(χ=10) and smoothed (Autodesk Meshmixer); i.e. see Fig. 2(c). The segmentation represents the lumen of the cerebral aqueduct and was offset by 3 mm to create a hollow model with sufficient wall thickness to provide structural rigidity. After scaling up the model, the resulting inner mean radius and length were $\overset{ˆ}{a}=1.3\;cm$ and =15.8cm, respectively, yielding an aspect ratio $\overset{ˆ}{a}/L=0.082$. The length of the real geometry was determined using the criterion defined in our previous study [31], where the rostral and caudal ends of the aqueduct were identified as the locations where the aqueduct cross-sectional area A increases by 50% from its mean value. The scaled model printed for this study included some additional portion of the third and fourth ventricles seen in the red highlighted segmentation of the MR image in Fig. 2(a). A detailed view of the canal geometry is included as an inset in Figs. 5(a) and 6(a), to be discussed later.

For both geometries the connecting elements, representing the aqueduct, were printed on a Form 3 Stereolithography (SLA) resin desktop printer using a rigid clear resin (Formlabs). Printed parts were post-processed in accordance with the manufacturer instructions. The longer geometries were printed in two separate pieces due to the size limitation of the printer. Following post-processing, both pieces were joined using UV resin welding and epoxy resin after full cure. The models have flange attachments with a silicone O-ring which are secured to the reservoirs by screws and liquid PTFE (teflon) to avoid leakage.

As mentioned above, the experiments were performed using as working fluid mixtures of glycerol and water at different concentrations, resulting in different kinematic viscosities, as needed to establish the periodic motion with the desired values of α. A single viscosity solution would not have been able to accommodate the limited range of the pump frequency, stroke volume and pressure sensor limits (see the discussion in Appendix). Because of the large viscosity of the working fluid, additional care was needed when filling and purging the system to avoid trapped air bubbles in the glycerol-water mixture.

The periodic volumetric flow rate Q(t) considered in the experiments included a simple sine wave $Q=\frac{1}{2}V_{s}\omega sin\;(\omega t)$ and a physiologically correct waveform determined using PC-MRI measurements in a healthy 25-year-old female subject [31]. In generating the periodic signal, the pulsatile pump uses as input the prescribed piston position, a zero-averaged signal that must start and end at the same value. The sinusoidal waveform is supplied as one of the default options in the pump software. For the realistic waveform, the piston position was determined using a trapezoidal cumulative numerical integration of the PC-MRI flow rate signal, and the result was scaled according to the manufacturer instructions to ensure the correct performance of the pump. The flow rate is adjustable within the limits of the pump, including frequencies in the range 3–200 BPM and stroke volumes in the range 0–180 mL/stroke.

## 4. Data acquisition

A wet/wet differential bidirectional pressure transducer (OMEGA Engineering) connected to the reservoirs was used to take measurements of Δp(t) (device specifications can be found in Table 1). Since the cross-section of the reservoirs is much larger than that of the aqueduct, the velocities in the reservoirs are much smaller than those found in the aqueduct, so that the pressure in each container is nearly uniform. As a result, the value of Δp is largely independent of the specific sensorplacement locations, provided that they are selected to be away from the entrance/exit regions. As indicated in Fig. 2(b), in our experiments the sensors were placed at the top of the reservoirs near the corners, where the fluid motion is negligible.

The system and pressure lines were checked to be bubble free. The pressure difference Δp(t) was measured with a sampling frequency of 80 Hz. To synchronize Δp(t) with Q(t) simultaneous recordings of instantaneous piston position were acquired using the pump’s voltage output. An Arduino Mega micro-controller, with an internal 10 bit ADC, was used to convert the voltage output to a digital signal, which was transmitted via a serial connection to a portable computer, where an open source serial data logger was used to time-stamp and save the data. The voltage was converted to piston position according to the manufacturer’s manual instructions. The sampling frequency of the analog output is 2500 Hz. The differences observed between the prescribed input piston position and the piston position computed from the pump’s voltage output were smaller than 5%, in agreement with the manufacturer’s stated waveform accuracy (i.e. <4% of stroke volume at 70 BPM and <5% of stroke volume at 200 BPM).

An experiment using a given glycerol-water mixture started by setting the corresponding amplitude-normalized wave form. Frequency values were selected in the range 0.15Hz≤ω/(2π)≤1.17Hz to provide the desired value of $\alpha ={\left(\omega_{E}{\overset{ˆ}{a}}_{E}^{2}/v_{E}\right)}^{1/2}$, with the kinematic viscosity of the mixture evaluated at the laboratory temperature (21.5 °C). Corresponding frequencies are listed in Table 2 for the different experiments reported below. Note that the expected departures of the working-fluid viscosity $v_{E}$ from the target value, of about 3% for the ±1 °C temperature variations measured between different experiments, may lead to errors ≲ 2% in the evaluation of the Womersley number.

With the pump running at constant frequency, pressure measurements were taken in subsequent tests for increasing values of the stroke volume $V_{s}=\pi {\overset{ˆ}{a}}^{2}L_{s}$, an input variable that could be adjusted manually to cover the range $0.5\leq L_{s}/L\leq 1.5$ in increments of $\Delta \left(L_{s}/L\right)=0.1.$ Each test comprised a total of 40–50 cycles. A complete list of measurements obtained is given in Table 2. For most frequencies, the measurements reported were taken during a single test, the exception being the cases indicated in bold in the table, for which measurements were made three separate times.

All signal processing was performed using a custom routine in MATLAB (Version R2019a; MathWorks). Signals were aligned using the timestamps of each recording. The pressure signal was filtered to eliminate noise and then zero-averaged. Pressure measurements from the sine wave flow rate were low-pass filtered at five times the selected frequency $\omega_{E}$, while the results corresponding to the subject-specific waveform were low-pass filtered at eight times $\omega_{E}$, as needed to retain sufficient high-frequency content. The piston-position signal, needed to synchronize the flow rate with the pressure difference, is subsampled to the same frequency as the pressure signal (80 Hz). This signal reading would produce some cases with sudden spikes much larger than the average peak value (>100x) which were detected using a Hampel filter and smoothed. The piston-position signal was differentiated to obtain the flow rate that was then low-pass filtered. The results were appropriately nondimensionalized according to

(10) $$\Pi (\tau )=\frac{\Delta p}{\rho_{E}\omega_{E}^{2}L_{E}L_{s_{,E}}}\;\text{and}\;\bar{Q}\left(\tau \right)=\frac{Q}{\omega_{E}\pi {\hat{a}}_{E}^{2}L_{s,E}},$$

where $\tau =\omega_{E}t$.

The above time-dependent functions were used to compute different quantities of interest. Time-averaged magnitudes of the pressure difference $\langle \left|\Pi \right|\rangle =\int_{0}^{2\pi }\left|\Pi \right|\text{d}\tau /\left(2\pi \right)$ were calculated using the trapezoidal rule. Another important quantity, with potential clinical relevance, is the phase lag between Π(τ) and $\overset{-}{Q}(\tau )$. Its definition is not unique. One could for example define the phase lag based on the relative position of the maxima or minima of the two signals within a given cycle, but with this definition the resulting values were found to be very sensitive to changes in the waveforms. A more robust approach, employed here, involves use of the spectral domain. Thus, Fast Fourier Transform (FFT) was used to determine the fundamental mode and its corresponding phase for each variable, i.e.

$$ℱ[(\overset{-}{Q}(\tau ))]\left(\omega_{0}\right)=\left|\overset{-}{Q}\left(\omega_{0}\right)\right|e^{i\varphi_{Q}}$$

$$ℱ[(\Pi (\tau ))]\left(\omega_{0}\right)=\left|\Pi \left(\omega_{0}\right)\right|e^{i\varphi_{\Pi }},$$

with the phase lag determined from the difference between both phases $\varphi =\varphi_{\Pi }-\varphi_{Q}$. For definiteness, φ is defined in the range -π≤φ≤π, so that a positive value indicates that the pressure difference peaks before the flow rate.

In processing the experimental results for given values of α and $L_{s}/L$, outliers were identified as those whose values of $\langle \left|\Pi \right|\rangle$ fall either below the 5th percentile or above the 95th percentile. The remaining experimental values were used to evaluate the mean and standard deviation of $\langle \left|\Pi \right|\rangle$ and φ, with results given in Figs. 4–6. The experimental results corresponding to the canonical model are to be compared with predictions obtained using a previously published flow model [30].

## 5. Results and discussion

**Fig. 3.**
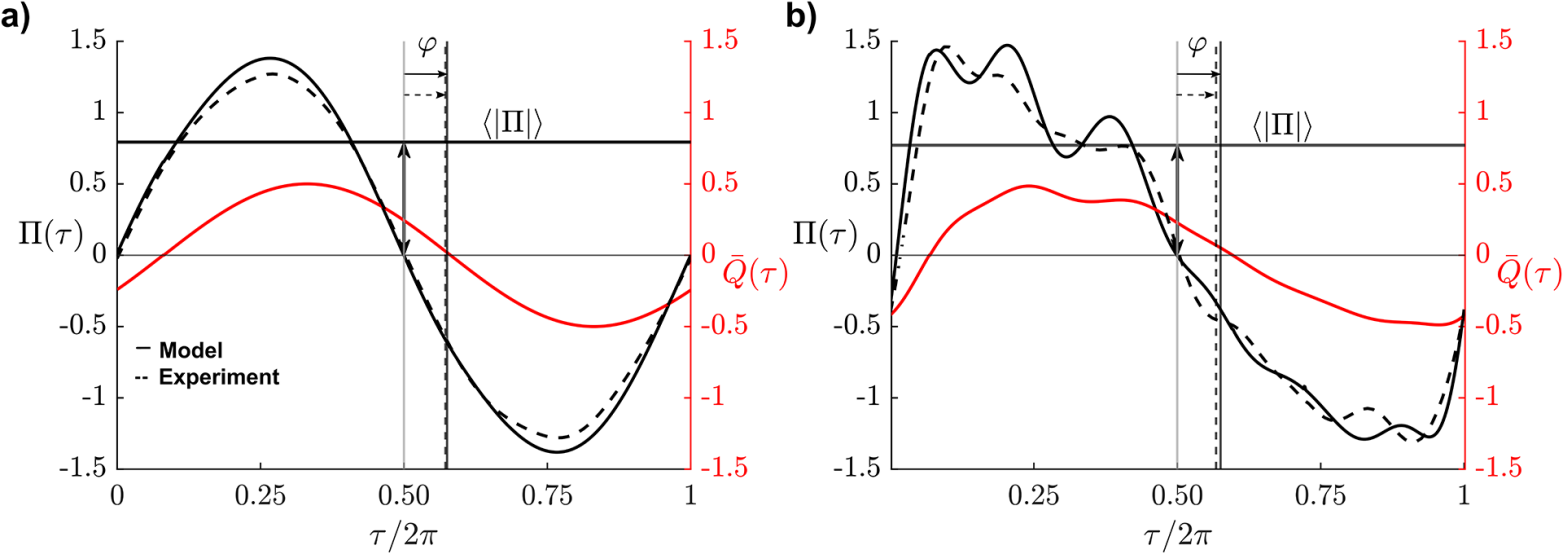
Dimensionless interventricular pressure Π(τ) corresponding to α=2 and $L_{s}/L=1$ as determined from the previously published mathematical model [30] (solid lines) and from the experimental measurements (dash lines) for the cylindrical aqueduct $\left(a/L=0.062\right.)$ with (a) a sinusoidal flow rate $\overset{‾}{Q}=\frac{1}{2}sin\;\tau$ and with (b) the PC-MRI flow rate.

Pressure readings were obtained for the geometries and parametric values indicated in Table 2. Resulting dimensionless pressure waveforms $\Pi (\tau )=\Delta p/\left(\rho \omega^{2}L_{s}L\right)$ corresponding to sinusoidal and physiologically correct flow rates for α=2 and $L_{s}/L=1$ are shown as dashed curves in Fig. 3 for the canonical geometry, with corresponding model predictions represented with solid lines. As shown in Fig. 3(a), for the sinusoidal flow rate, given by $\overset{-}{Q}=\frac{1}{2}sin\;(\tau )$ in dimensionless form, the associated differential pressure is found to be nearly sinusoidal. By way of contrast, the physiologically correct flow rate, considered in Fig. 3(b), induces a markedly anharmonic transmantle pressure Π(τ) exhibiting three peaks that are reminiscent of the percussion, tidalwave, and dicrotic-wave peaks characterizing the intracranial pressure waveform [40].

The theoretical predictions are in reasonably good agreement with the experimental measurements. The resulting time-averaged fluctuation amplitudes are $\langle \left|\Pi \right|\rangle =0.88$ (model) and $\langle \left|\Pi \right|\rangle =0.79$ (experiment) for the sinusoidal flow rate of Fig. 3(a) and $\langle \left|\Pi \right|\rangle =0.88$ (model) and $\langle \left|\Pi \right|\rangle =0.77$ (experiment) for the PC-MRI flow rate of Fig. 3(b), with corresponding phase lags being φ=0.47 (model) and φ=0.45 (experiment) in Fig. 3(a) and φ=0.47 (model) and φ=0.42 (experiment) in Fig. 3(b). The departures observed are consistent with the errors, of order $\overset{ˆ}{a}/L$, that are inherent in the model approximations [30]. As can be seen, the shape of the pressure wave is closely reproduced by the model when the flow rate is sinusoidal, while for the physiologically correct flow rate the model tends to amplify somewhat the high-frequency peaks. This feature of the model prediction is attributable to the quasi-steady approximation employed in analyzing the entrance regions [30], which implies an instantaneous pressure response to a rapid flow-rate fluctuation.

**Fig. 4.**
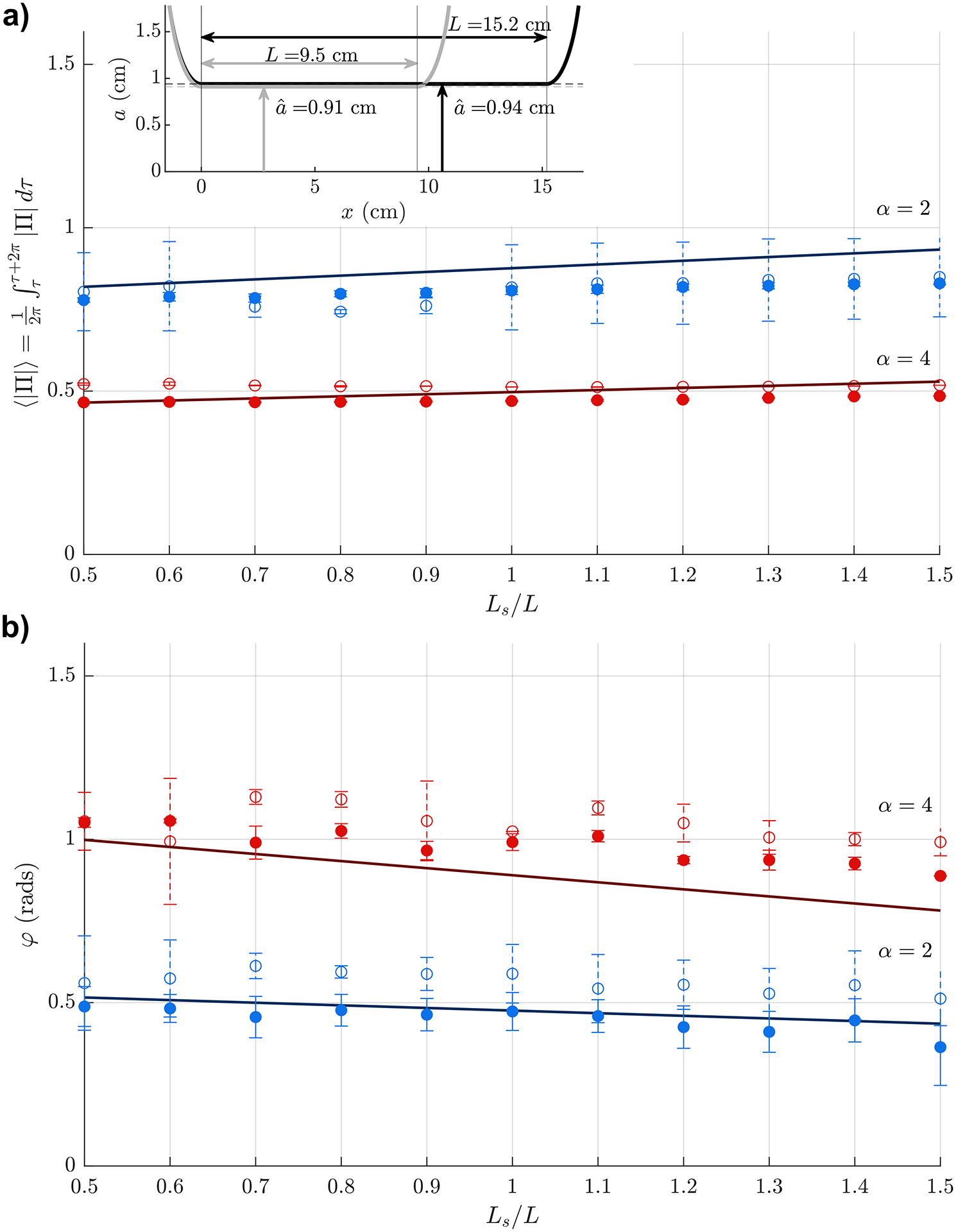
The variation with $L_{s}/L$ of (a) the time-averaged magnitude of the pressure difference $\langle \left|\Pi \right|\rangle$ and (b) the phase lag φ for α=2 (blue) and α=4 (red) determined experimentally using a sinusoidal flow-rate waveform, with the aqueduct represented by a cylindrical tube of aspect ratio $\overset{ˆ}{a}/L=0.096$ (hollow symbols) and $\overset{ˆ}{a}/L=0.062$ (solid symbols). Error bars are used to indicate the standard deviation of the experimental data. Theoretical predictions obtained with use of our reduced-flow model [30] are represented as solid lines.

Fig. 4 represents the variation with $L_{s}/L$ and α of the dimensionless time-averaged pressure fluctuations $\langle \left|\Pi \right|\rangle$ and accompanying phase lags φ determined in experiments using the two circular cylinders represented in the bottom-left subplot of Fig. 2(c), with results identified by either hollow markers $\left(\overset{ˆ}{a}/L=0.096\right)$ or filled markers $\left(\overset{ˆ}{a}/L=0.062\right)$. For each set of values of $\overset{ˆ}{a}/L,L_{s}/L$ and α, the values of $\langle \left|\Pi \right|\rangle$ and φ reported in the figure are determined by averaging measurements corresponding to different frequencies and three different glycerol-water mixtures, as indicated in Table 2. Error bars indicate the standard deviation of the measurements. Following the dimensional analysis presented earlier, the results are plotted for values of the reduced stroke length in the range $0.5\leq L_{s}/L\leq 1.5$ and two different values of the Womersley number, namely, α=2 (blue symbols) and α=4 (red symbols). Results acquired for α=3 are not included in the plot to avoid excessive cluttering. Theoretical predictions obtained with the mathematical model derived in [30], independent of $\overset{ˆ}{a}/L$, are represented by solid lines.

The results in Fig. 4 support the parametric characterization of the flow, in that there is good agreement between the different experiments when the results are represented in the dimensionless form identified in (4). The dispersion of the experimental data, measured by the error bars indicating the standard deviation, is partly attributable to the experiment-to-experiment variability of the mixture properties, associated with changes in composition and temperature, and partly attributable to the pressure transducer accuracy. The comparison between the hollow and solid symbols reveals that the dependence of the flow on the aqueduct aspect ratio is weak, in agreement with theoretical considerations pertaining to slender flows [30], especially in connection with the mean value of the interventricular pressure$\langle \left|\Pi \right|\rangle$, which is almost identical for the experiments conducted with $\overset{ˆ}{a}/L=0.096$ and $\overset{ˆ}{a}/L=0.062$. Somewhat larger differences are found in the measurements of the phase lag φ corresponding to the two cylindrical models. The results for $\overset{ˆ}{a}/L=0.062$ (solid symbols) are seen to lie closer to the values predicted with the reduced model, as is to be expected, since the model exploits the asymptotic limit $\overset{ˆ}{a}/L\ll 1$. The departures between the theoretical predictions and the experimental results, on the order of 10% for $\langle \left|\Pi \right|\rangle$ and 20% for φ, are consistent with the errors of order $\overset{ˆ}{a}/L$ present in the theoretical development [30].

The analysis reveals that both $\langle \left|\Pi \right|\rangle$ and φ depend weakly on the dimensionless stroke length, variations remaining below about 10% over the entire range $0.5\leq L_{s}/L\leq 1.5$ explored in the figure. By way of contrast, the dependence of the results on α is much more pronounced. As can be seen in Fig. 4(a), as the Womersley number increases from α=2 to α=4, the pressure drop decreases by about 40%. In understanding this decrease, one must bear in mind that the pressure drop is partly due to the nearly inviscid acceleration occurring at the tube entrance, leading to a local pressure drop of order $\rho \omega^{2}L_{s}^{2}$, and partly due to the viscous shear stresses acting on the tube wall, which lead to a longitudinal pressure drop of order $\mu \omega L_{s}L/{\overset{ˆ}{a}}^{2}$, where μ=ρν denotes the dynamic viscosity. While only the first contribution is present in the inviscid limit α≫1 [30], in the general case α~1 one needs to account for the additional effect of viscous forces, whose relative contribution to the total pressure drop, smaller for larger α, can be seen to scale with $\alpha^{-2}{\left(L_{s}/L\right)}^{-1}$ [30], thereby explaining the pressure-drop reduction shown in Fig. 4(a).

The sharp increase in the phase lag with increasing α seen in Fig. 4(b) can be similarly explained by noting that the phase lag between the interventricular pressure and the flow rate is mainly a result of the interplay of the different forces entering in the momentum equation. In flows at low Womersley numbers, there exists a balance between viscous and pressure forces, so that the associated flow rate Q(t) is in phase with Δp. In contrast, in the opposite limit α≫1, the flow rate and the interventricular pressure can be expected to be in quadrature, as follows from a balance between local acceleration and pressure gradient. The results in Fig. 4(b) are therefore consistent with the expected transition from φ=0(α≪1) to φ=π/2(α≫1).

**Fig. 5.**
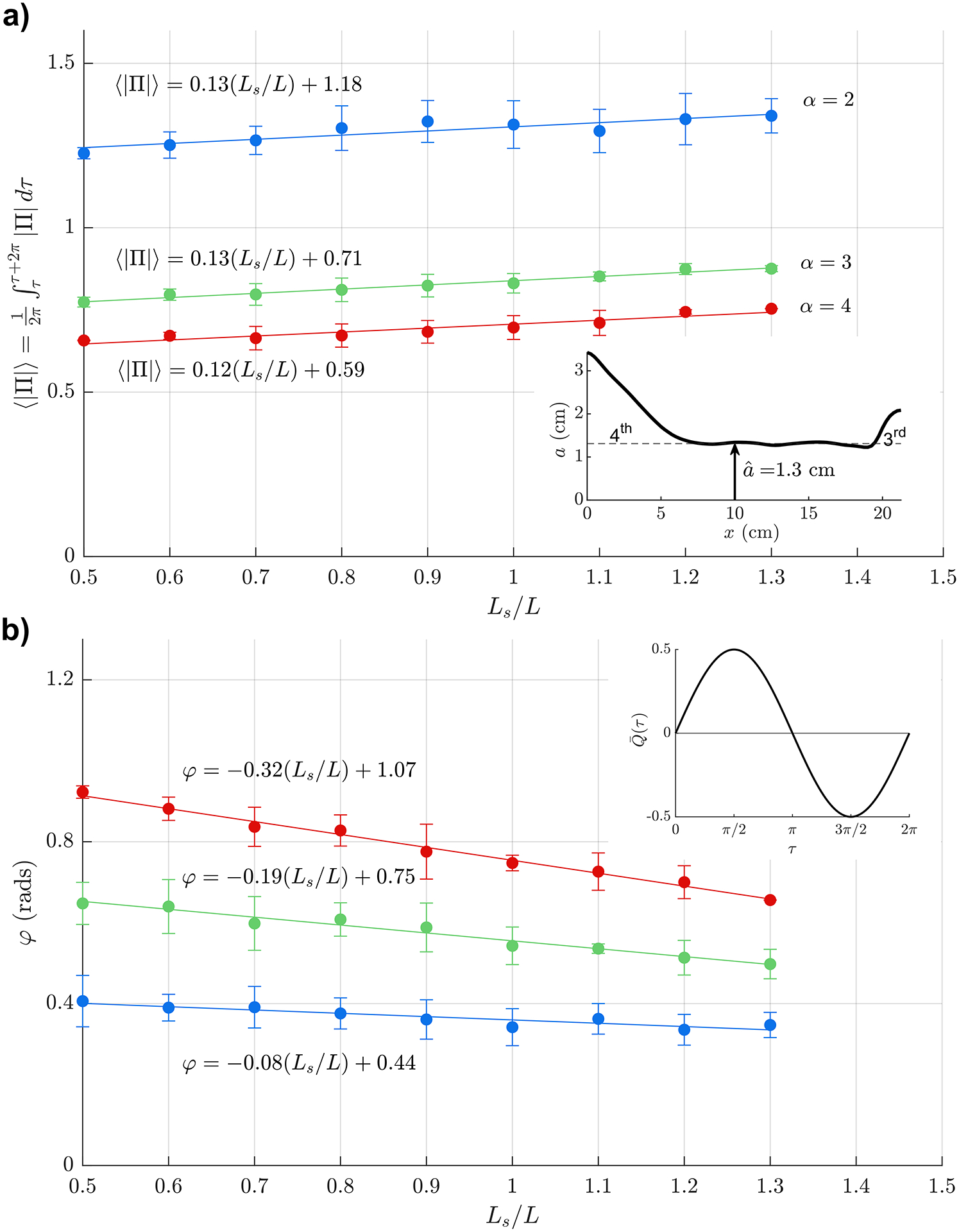
The variation with $L_{s}/L$ of (a) the time-averaged magnitude of the pressure difference $\langle \left|\Pi \right|\rangle$ and (b) the phase lag φ for α=(2,3,4) (blue, green and red, respectively) as obtained experimentally using an anatomically correct aqueduct shape together with a sinusoidal flow rate. Symbols represent values computed by averaging the experimental measurements corresponding to three different glycerol-water mixtures and different flow frequencies, as described in Table 2, with error bars used to indicate the standard deviation of the measurements. Also shown in the figure are the lines of best fit that approximate the experimental data corresponding to each value of α.

**Fig. 6.**
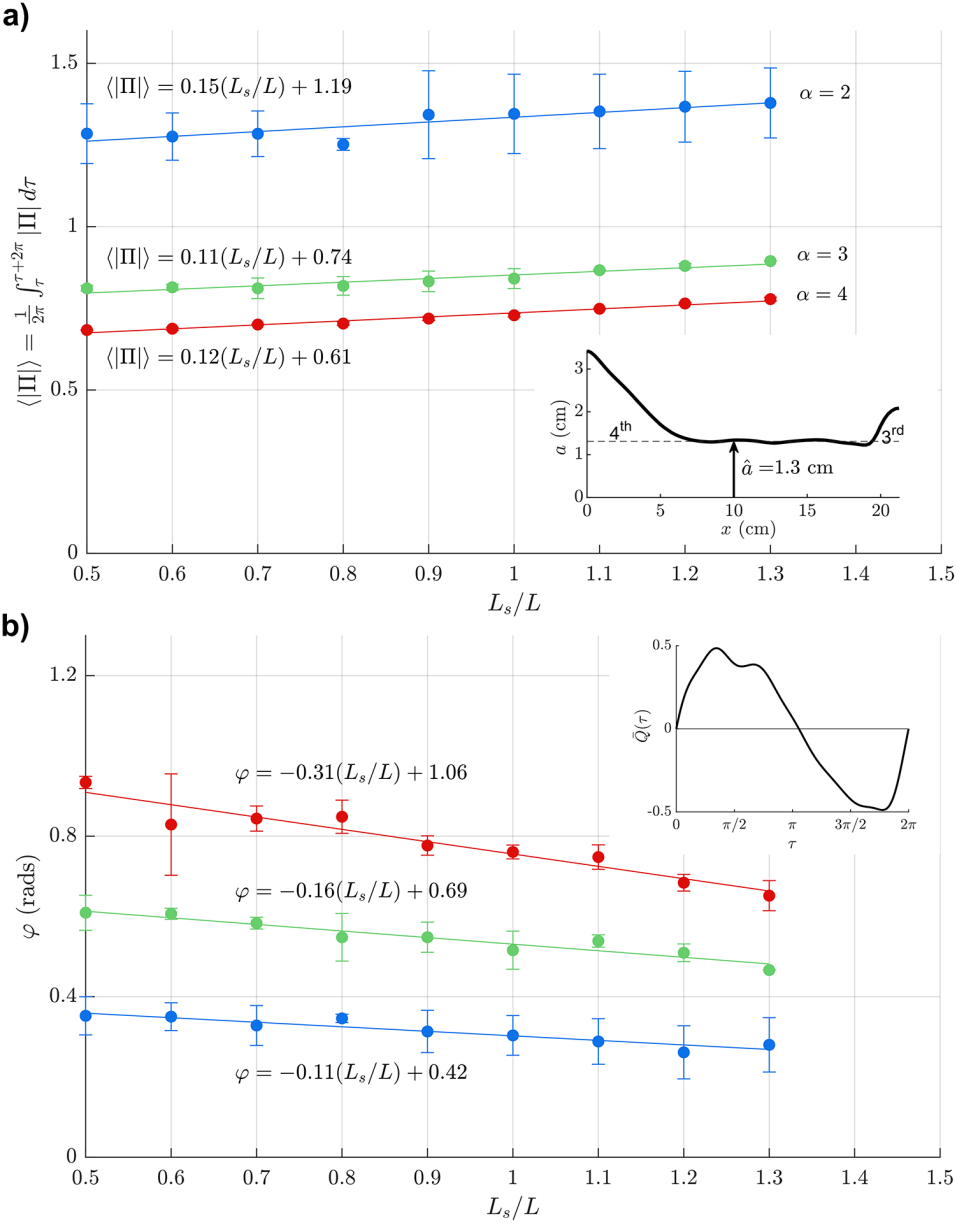
The variation with $L_{s}/L$ of (a) the time-averaged magnitude of the pressure difference $\langle \left|\Pi \right|\rangle$ and (b) the phase lag φ for α=(2,3,4) (blue, green and red, respectively) as obtained experimentally using an anatomically correct aqueduct shape together with the physiologically correct flow rate represented in the inset of the lower panel. Symbols represent values computed by averaging the experimental measurements corresponding to three different glycerol-water mixtures and different flow frequencies, as described in Table 2, with error bars used to indicate the standard deviation of the measurements. Also shown in the figure are the lines of best fit that approximate the experimental data corresponding to each value of α.

Measurements taken using the anatomically correct model are presented for both the sinusoidal (Fig. 5) and the physiologically correct (Fig. 6) flow rates Q(t). As in Fig. 4, error bars indicate the standard deviation of the measurements, performed using three different glycerol-water mixtures and different flow frequencies, listed in Table 2. For this geometry, the large pressure variations associated with the experiments at large stroke volumes were found to compromise the structural integrity of the reservoirs, so that the experiments were restricted to values of $0.5L_{s}/L\leq 1.3$. For each value of α, the measurements display a nearly linear variation with $L_{s}/L$ that can be accurately described using a line of best fit, with associated equations given in the figure.

Effects of canal anatomy on pressure drop can be assessed by comparing the results shown in Figs. 4 and 5, both corresponding to a sinusoidal flow rate. As can be seen, when written in dimensionless form, the interventricular pressure measured in the anatomically correct experiments is noticeably larger than that of the circular cylinders, a result attributable to the additional pressure loss occurring in the nozzle-like entrance regions of the third and fourth ventricles, which are included in the realistic model, as indicated in the inset of Fig. 5(a). In principle, the presence of these regions could be accounted for in defining the canal length, leading to larger values of L and correspondingly smaller values of$\langle \left|\Pi \right|\rangle$, as follows from its definition (4), thereby possibly improving agreement between the canonical and anatomically correct results.

The comparison between Figs. 5 and 6 reveals that, while the waveform of the interventricular pressure is drastically different for the sinusoidal and physiologically correct flow rates, as seen in Fig. 3, the differences in the mean pressure fluctuation $\langle \left|\Pi \right|\rangle$ remain smaller than 5% for all values of α and $L_{s}/L$. The differences in the phase lag are also very small, as seen in the lower plots, indicating that, for many quantitative purposes the flow rate can be represented by a simple sinusoidal function, as done in earlier analyses [19].

Of particular interest for quantitative purposes are the results shown in Fig. 6, correspondingly to an anatomically correct aqueduct geometry and a physiologically correct flow rate. Fig. 6(b) shows the phase difference φ between Δp(t) and Q(t), a metric with potential clinical application [26]. As expected, the resulting value, weakly dependent on $L_{s}/L$, is found to increase as flow acceleration becomes more pronounced with increasing $\alpha =\overset{ˆ}{a}\sqrt{\omega /v}$ (i.e. for increasing flow frequency and aqueduct radius). On the other hand, the value of $\langle \left|\Pi \right|\rangle$ shown in Fig. 6(a) and the associated linear fits given in the figure for each value of α can be used in (4) to provide

(11) $$\frac{\omega }{2\pi }\int_{t}^{t+2\pi /\omega }\left|\Delta p\right|dt=\left\langle \left|\Pi \right|\right\rangle \rho \omega^{2}L\frac{V_{s}}{\pi {\overset{ˆ}{a}}^{2}},$$

which can be useful in estimating the mean value of the transaqueductal pressure fluctuation from MRI measurements of the stroke volume $V_{s}$ and the cerebral-aqueduct anatomy, the latter entering through the values of L and $\overset{ˆ}{a}=\int_{0}^{L}\;\sqrt{A/\pi }dx/L$.

The above expression (11) is useful in discussing the clinical relevance of the stroke volume, a metric previously proposed as a predictor for shunt response in patients with normal pressure hydrocephalus [6]. Compared with normal subjects, NPH patients are known to have larger stroke volumes [41], which, according to (11), would be indicative of augmented interventricular pressure fluctuations, a reasoning that implicitly assumes that the aqueduct radius remains constant. However, enlargement of the aqueduct leads to a drastic reduction in the transaqueductal pressure, both because of the direct proportionality $\langle \left|\Delta p\right|\rangle \propto {\hat{a}}^{-2}$ present in (11) and also because $\langle \left|\Pi \right|\rangle$ decreases with increasing $\alpha =\overset{ˆ}{a}\sqrt{\omega /\nu }$, as shown in Fig. 6(a). As a result, patients with simultaneous increased stroke volume and enlarged aqueduct cross-section may exhibit normal values of Δp, as revealed in *in vivo* studies [21]. Clearly, future investigations addressing this issue can benefit from the simple quantitative description provided in (11).

It is worth mentioning that, since in most cases the largest spatial pressure drop occurs across the aqueduct, Eq. (11) can be applied in general to quantify the mean transmantle pressure from non-invasive MRI measurements of the stroke volume. In using the equation, one should keep in mind that the transmantle pressure may differ significantly from the transaqueductal pressure in pathologic cases involving exceedingly enlarged aqueducts, stenosed foramina of Monro or other obstructions in the ventricular flow path; for these cases, Eq. (11) would give an inaccurate representation of the transmantle pressure.

## 6. Conclusions and future prospects

Experiments employing a scaled physical model of the cerebral aqueduct have been used to characterize the relation between the flow rate Q(t) and the interventricular pressure difference Δp(t), thereby complementing previous experimental efforts [35–38]. The development exploits the fact that Δp can be determined by investigating the flow through the aqueduct, without specific knowledge of the brain compliance, which is, however, essential for determining the temporal intracranial pressure fluctuations [42]. The focus has been on the determination of the time-averaged magnitude of the interventricular pressure $\langle \left|\Delta p\right|\rangle$ and of the phase difference φ between Δp(t) and Q(t), both quantities potentially having clinical interest in connection with NPH [25,26]. Dimensional analysis has been used to simplify the parametric dependence, leading to the reduced functional dependence identified in (4). A first set of experiments using two circular cylinders showed that the flow is fairly independent of the aspect ratio $\overset{ˆ}{a}/L$, so that the solution depends mainly on two parameters, namely, the Womersley number α and the dimensionless stroke length $L_{s}/L$. The results have been validated through comparisons with theoretical predictions obtained with a previously derived reduced description exploiting the slenderness of the flow [25]. Measurements corresponding to an anatomically correct aqueduct geometry are acquired over ranges of α and $L_{s}/L$ describing the flow in the aqueduct of the healthy subject. The mean interventricular pressure fluctuation $\langle \left|\Delta p\right|\rangle$ determined using a physiologically correct flow rate, shown in Fig. 6(a), can be used together with the simple formula given in (11) to estimate the mean transaqueductal pressure difference, which can be used as a reasonable proxy for the transmantle pressure difference.

Future investigations could benefit from improvements to the experimental apparatus. For instance, because of the volumetric size and available compliance of the experimental setup, the experiments reported above were limited on the range of stroke volumes and frequencies applied to the system, with additional limitations arising from the available pressure transducer. A lower frequency and/or lower stroke volumes incurred a larger noise contribution in the measurements. This was partially remedied through the use of different glycerol-water viscosity values and our attempt to balance the stroke volume and frequency. Additional potential sources of error include the presence of air bubbles within the main setup and possible inhomogeneities in the glycerol-water mixtures.

The characterization of the phase lag on the basis of the fundamental frequency was found to be more robust than measurements based on cross-correlation or peak-to-peak time difference, which is partly due to the occurrence of signal jitter. Since the resolution of the frequency analysis increases as the number of periods sampled increases, future experiments should be recorded for longer duration, to include more periods and correspondingly reduce associated errors. Additional systematic errors induced by the time-stamp alignment, which is on average about 1 ms (<5% error), can be minimized by sampling over many cycles and performing repeated experiments, as done in the present study.

The novel setup enables investigation of various input parameters (signal waveform, fluid viscosity, aqueduct geometry), allowing us to measure the corresponding effects on the interventricular pressure. Future studies should consider the systematic variation of these parameters to understand certain diseases. Specifically, this experimental setup could be used to investigate the connection between changes in the frequency composition of the flow-rate signal, reported for example in [43], and the associated transmantle pressure. Also of interest are investigations of the relevance of the phase lag φ as a potential new marker of interest [26] as well as studies addressing diseases that result in altered cerebrospinal fluid flow in the aqueduct such as NPH.

## Figures and Tables

**Table 1:** Differential pressure transducer configuration.

Name	Description
Transducer material	316L stainless steel
Process fitting	1/4–18 NPT female
Pressure type	Diff. Wet/Wet bidirectional
Range unit	in-H2O (4 °C)
in-H2O	10
Pressure transducer output	USB (high-speed)
Pressure transducer accuracy	±0.08% B.S.L.
Electrical termination	Cable (2 m, 6ft)
Temperature range	18 to 85 °C (−0 to 185 °F)
Thermal accuracy: Zero shift/Span shift	±0.80%/±0.60%

**Table 2:** List of experiments conducted for each geometry and viscosity value with corresponding frequency ω/(2π) (expressed here in beats per minute) and sampled range of dimensionless stroke length (increment $\Delta L_{s}/L=0.1$)

	Canonical (short)		Canonical (long)		Real geometry	
*Glycerol-water (84–16 volume) v* = 1.05 × 10^−4^ m^2^/s						
*α*	*ω/2π* (BPM)	*L*_*s*_ */L*	*ω/2π* (BPM)	*L*_*s*_ */L*	*ω/2π* (BPM)	*L*_*s*_ */L*
**2**	**48**	**0.5**–**1.5**	**45**	**0.5**–**1.5**	**24**	**0.5**–**1.3**
3	–	–	–	–	53	0.8–1.1
4	–	–	–	–	–	–
*Glycerol-water (80–20 volume) v* = 6.82 × 10^−5^ m^2^/s						
2	31	0.5–1.5	29	0.5–1.4	15	0.8–1.1
**3**	**71**	**0.5**–**1.5**	**66**	**0.5**–**1.5**	**35**	**0.5**–**1.3**
4	126	0.5–0.8	–	–	62	0.5–1.1
*Glycerol-water (74–26 volume) v* = 3.83 × 10^−5^ m^2^/s						
2	18	0.5–1.5	17	0.5–1.5	9	0.5–1.3
3	40	0.5–1.5	37	0.5–1.5	19	0.5–1.3
**4**	**71**	**0.5**–**1.5**	**66**	**0.5**–**1.5**	**35**	**0.5**–**1.3**

## Data Availability

Data will be made available on request.

## Appendix. Mathematical basis for the selection of the working fluid

The transaqueduct pressure $\Delta p=p_{e}-p_{4}$ measured in the experiments changes from positive to negative, with peak values that are about $\Delta p_{\text{peak}}≃2\langle |\Delta p|\rangle$, as can be inferred from the dimensionless plots in Fig. 3. Correspondingly, one can use (4) to write

(A.1)
$$\frac{\Delta p_{\text{peak}}}{\rho \omega^{2}L^{2}}=2\left(L_{s}/L\right)\langle \left|\Pi \right|\rangle \left(\alpha ,L_{s}/L\right),$$

where the Womersley number α and the dimensionless stroke length $L_{s}/L$ vary in the range

$$2\leq \alpha \leq 4\;\text{and}\;0.5\leq L_{s}/L\leq 1.5.$$

The plot provided in our computational model [30] shows that $\langle \left|\Pi \right|\rangle$ is nearly independent of $L_{s}/L$, a result confirmed by the experimental results presented in Figs. 4 and 5, so that, to a good approximation, one can use

(A.2)
$$\langle \left|\Pi \right|\rangle =\left\{\begin{array}{ll} 0.8, & \text{ for }\alpha =2\\ 0.6, & \text{ for }\alpha =3\\ 0.5, & \text{ for }\alpha =4. \end{array}\right.$$

to evaluate$\langle \left|\Pi \right|\rangle$.

To stay within the operational limits of the pressure sensor, the pressure difference $\Delta p_{\text{peak}}$ has to satisfy

(A.3)
$$500\;Pa<\Delta p_{\text{peak}}<1500\;Pa.$$

To determine the range of viscosity values needed to satisfy this constraint for each value of α, one can use (A.1) to write

(A.4)
$$\omega ={\left[\frac{\Delta p_{\text{peak}\;}}{2\langle \left|\Pi \right|\rangle \rho L^{2}\left(L_{s}/L\right)}\right]}^{1/2}$$

and substitute the result into (5) to yield

(A.5)
$$v=\frac{a^{2}}{\alpha^{2}}{\left[\frac{\Delta p_{\text{peak}\;}}{2\langle \left|\Pi \right|\rangle \rho L^{2}\left(L_{s}/L\right)}\right]}^{1/2}.$$

If we choose a target value of $\Delta p_{\text{peak}}=1000\;Pa$ and use

(A.6)
$$\rho =1.2\cdot 10^{3}\;kg/m^{3},\;L=0.15\;m,\;\text{and}\;a=0.01\;m.$$

as representative values, then straightforward evaluation of (A.5) provides the value of v corresponding to given values of α and $L_{s}/L$, yielding

(A.7)
$$\alpha =2\;:\;v=\left\{\begin{array}{ll} 1.70\times 10^{-4}{\;m}^{2}/s, & \text{for }L_{s}/L=0.5\\ 9.82\times 10^{-5}{\;m}^{2}/s, & \text{for }L_{s}/L=1.5. \end{array}\right.$$

(A.8)
$$\alpha =3\;:\;v=\left\{\begin{array}{ll} 8.73\times 10^{-5}{\;m}^{2}/s, & \text{for }L_{s}/L=0.5\\ 5.04\times 10^{-5}{\;m}^{2}/s, & \text{for }L_{s}/L=1.5. \end{array}\right.$$

and

(A.9)
$$\alpha =4\;:\;v=\left\{\begin{array}{ll} 5.38\times 10^{-5}{\;m}^{2}/s, & \text{for }L_{s}/L=0.5\\ 3.11\times 10^{-5}{\;m}^{2}/s, & \text{for }L_{s}/L=1.5. \end{array}\right.$$

for the two limiting values of $L_{s}/L$. For each value of α, ratios of glycerol-water were chosen to give mixture viscosities within each of the above limits at a steady controlled temperature of 21.5 °C.
